
JOURNAL INFORMATION
==============================
NLM Title Abbreviation: Buffalo Med J
NLM Journal ID: 0027534

Buffalo Medical Journal

ISSN: 1040-3817
Publisher: Biomedical Journal Digitization Project

ARTICLE INFORMATION
==============================
PMCID: PMC8768624
PMID: 36888435
Article version: 1
Subjects: Miscellany

Miscellany

Print publication date: 1908 Apr
Volume: 63
Issue: 9
First page: 558
Last page: 558
License: This work is free of known copyright restrictions. For more information, please see PMC Back Issue Digitization.
License URL: https://creativecommons.org/publicdomain/mark/1.0/

==============================

Annual Report of the Surgeon-General of the Public Health and Marine Hospital Service of the United States. For the fiscal year, 1907. Walter Wyman, M.D., Surgeon-General. Washington: Government Printing Office.

Progressive Medicine, Vol. X, March, 1908. A Quarterly Digest of Advances, Discoveries and Improvements in the Medical and Surgical Sciences. Edited by Hobart Amory Hare, M.D., Professor of Therapeutics and Materia Medica in the Jefferson Medical College of Philadelphia. Octavo, 284 pages. Lea Brothers & Co., Publishers, Philadelphia and New York. Per annum in four cloth-bound volumes, $9.00; in paper binding, $6.00, carriage paid to any address.

### MISCELLANY.

The State Civil Service Commission will hold examinations on April 18, 1908, for among others the following positions: Examiners in English and Sciences, Education Department, $720.00 to $900.00, also for temporary summer employment at $60.00 to $100.00 a month; Health Officer, Town of Richmond, Ontario county.

The last day for filing applications for these positions is April 11. Full information and application forms for any of these examinations may be obtained by addressing Charles S. Fowler, Chief Examiner, Albany.

The Bowery Mission, conducted by Louis Klopsch, has notified the governors of the several states that the mission can supply at a moment's notice any number of able-bodied men for unskilled labor to the farms and industrial centers where there is a marked dearth of labor. All that is required to secure such help is to pay transportation. Applications should be addressed John C. Earl, Free Labor Bureau, 92 Bible House, New York City.

Attention is called to the Alaska-Yukon-Pacific Exposition (*St. Louis Med. Review*), which will be held at Seattle, Washington, opening June 1, and closing October 15, 1909.

This exposition will be held in a section of the country where world's fairs are new and for that and many other reasons it is expected to be a success, beneficially, educationally, artistically and financially.

It is said that work on the grounds and buildings of the exposition is well under way and the management is determined to have everything in readiness by opening day. The purpose of the exposition, is to exploit Alaska and Yukon and the countries bordering on the Pacific Ocean.

FOR SALE.—Van Houten & Ten Broeck static machine with 12 revolving plates of 30 inches diameter, full set of electrodes and x-ray tubes. The machine can be seen at the office of Dr. Eugene Horton, Gluck Building, Niagara Falls, N. Y., or apply to Mrs. W. R. CAMPBELL, 34 Niagara St., Niagara Falls.
